# Understanding peer bullying and broader school aggression from a school psychology perspective: a Social-Ecological Model based on extracurricular activities, instructional capacity, and learning contexts

**DOI:** 10.3389/fpsyg.2026.1775082

**Published:** 2026-03-30

**Authors:** Abdurrahman İlğan, Servet Üztemur, Ali Gökalp

**Affiliations:** 1 Department of Educational Sciences, Faculty of Education, İzmir Democracy University, Izmir, Türkiye; 2 Department of Turkish and Social Sciences Education, Faculty of Education, Anadolu University, Eskişehir, Türkiye; 3 Department of Educational Sciences, Gaziantep University, Gaziantep, Türkiye

**Keywords:** extracurricular activities, misconduct behaviors, peer bullying, school aggression, school psychology, Social-Ecological Model

## Abstract

**Background:**

Peer bullying is increasingly recognized as a contextual issue shaped by the school environment. Although extracurricular activities are often considered protective, their association with school-level aggressive and misconduct behaviors, including peer intimidation-related behaviors, appears to depend on broader institutional factors rather than operating in isolation.

**Objective:**

Grounded in a social-ecological framework, this study investigates the relationship between the availability of extracurricular activities and school-level aggressive and misconduct behaviors, specifically examining the mediating role of school-level instructional barriers and the moderating role of learning-related hindrances.

**Methods:**

Cross-sectional data from the PISA 2022 database were analyzed. After handling missing data, the final analytic sample comprised 16,555 schools across 75 countries. A moderated mediation model was tested to examine the association between the availability of extracurricular activities and school-level aggressive and misconduct behaviors, with problems hindering the school’s instructional capacity as a mediator and phenomena hindering students’ learning as a moderator of the direct association.

**Results:**

Greater availability of extracurricular activities was associated with lower levels of school-level aggressive and misconduct behaviors, indirectly through reduced problems that hindered the school’s instructional capacity. In addition, the direct association between extracurricular activities and school-level aggressive and misconduct behaviors varied depending on the level of phenomena hindering students’ learning. These findings indicate that the relationship between extracurricular activities and aggression- and misconduct-related school problems is shaped not only by institutional barriers but also by the broader school learning context.

**Conclusion:**

The findings suggest that extracurricular activities are not standalone protective factors but operate within a broader school ecology. Their protective potential is partly explained by instructional capacity and depends on the level of learning-related hindrances within the school environment, highlighting the need for comprehensive school-wide strategies addressing broader aggression- and misconduct-related school problems, including peer bullying-related behaviors.

## 1 Introduction

Participation in extracurricular activities is a crucial factor with far-reaching positive effects on the development of young people. In contrast to school settings, these activities allow students to explore personal interests and express themselves in a safe environment (Berger et al., 2020). Extracurricular activities have a significant impact on students’ self-efficacy in specific domains, such as extroversion and help-seeking (Griffiths et al., 2021). Moreover, these activities have the potential to increase young people’s social competence, build team spirit, reduce depressive symptoms, improve mental health, improve academic performance, build stronger relationships, develop a sense of belonging, acquire socio-emotional skills, and promote long-term resilience and well-being (Abizada et al., 2020; Berger et al., 2020; Berger et al., 2021; O’Flaherty et al., 2022; Venkataraman and Dheivamani, 2021). Even in challenging life circumstances, these programs support individuals’ basic psychological needs, such as autonomy, competence, and social connectedness, to help them avoid risky behaviors. Furthermore, these supportive effects have lasting, positive effects that extend into adulthood (Décarpentrie et al., 2024). It has been observed that extracurricular activities strengthen an individual’s self-perception, and these positive effects are more pronounced among disadvantaged youth, particularly those from schools with low socioeconomic status (Blomfield and Barber, 2011). For individuals with disabilities, participation in extracurricular activities allows them to feel psychosocially stronger by reducing the risk of bullying (Haegele et al., 2020). However, because each extracurricular activity is unique and because students, teachers, and parents have different perceptions, it can lead to varied outcomes (Feldman and Matjasko, 2005). School-level aggressive and misconduct behaviors have been widely addressed as issues that negatively affect the well-being of school-age children and adolescents (Sheridan et al., 2022). The outcome examined in this study should be understood as a broader school-level aggression and misconduct construct that includes peer intimidation as one of its components, rather than as a narrow indicator of bullying alone. In this context, the relationship between participation in extracurricular activities and bullying tendencies, as well as exposure to bullying, is complicated, and the literature presents both protective and, in some cases, risk-increasing findings (Berger et al., 2021; Peguero, 2008; Riese et al., 2015; Sheridan et al., 2022).

Research shows that children who engage in both sports and non-sports activities are significantly less likely to bully (Fredricks and Eccles, 2006; Riese et al., 2015). Sports and organized activities, in particular, have been suggested as potential protective factors against bullying victimization and perpetration (Hong et al., 2023). Regular participation in high-quality after-school programs has been linked to decreased aggression toward peers and lower reports of inappropriate behavior (Vandell et al., 2022). It has also been proposed that extracurricular activities can promote prosocial (altruistic) behaviors, thereby serving as a protective factor against bullying and other forms of violence (Berger et al., 2021). Belonging to a prosocial peer network has been found to partially mediate the link between extracurricular involvement and lower depression levels, as well as higher school engagement (Fredricks and Eccles, 2005). However, it has been emphasized that combining extracurricular activities with unsupervised time may be associated with lower academic performance and social functioning in children, as well as potentially problematic behaviors such as aggression and inappropriate conduct (Vandell et al., 2022). From the perspective of bullying victimization, the relationship is even more complex. Some studies have shown that participating in athletic teams reduces the risk of verbal bullying victimization and tends to be protective overall (Sheridan et al., 2022). This may be explained by the fact that interscholastic athletes are perceived to have higher social status and physical strength, making them less vulnerable targets for potential aggressors (Broh, 2002; Peguero, 2008). However, it has been reported that participation in performing arts, service clubs, and activities in the “other” category increases the risk of verbal, relational, and physical bullying victimization; in fact, students in performing arts groups may be particularly vulnerable to all types of bullying (Elpus and Carter, 2016; Sheridan et al., 2022). Similarly, it has been observed that students who participate in three or more in-class activities or intramural sports are more likely to be bullied (Peguero, 2008), while participation in multiple extracurricular activities increases the likelihood of overall bullying victimization (Sheridan et al., 2022). This may be explained by factors such as students participating in these activities being perceived as weaker targets by potential aggressors (“smart” kids tend to be “picked on” more often) or spending more time at school, which provides more opportunities for bullying (Eisenberg et al., 2003; Juvonen et al., 2000; Peguero, 2008). On the other hand, greater participation in extracurricular activities is associated with more positive academic and psychological adjustment, and the diversity of activities is associated with a lower tendency to bully than uniform participation (Fredricks and Eccles, 2006; Mahoney et al., 2003; Riese et al., 2015). Demographic and contextual factors, such as gender, age, ethnicity, socioeconomic status, and school social norms, have been shown to influence these relationships significantly (Berger et al., 2021; Riese et al., 2015; Sheridan et al., 2022). In particular, participation in activities that violate gender norms (e.g., boys participating in female-dominated sports or girls participating in male-dominated sports) has been associated with victimization risk (Agnich et al., 2017). In the case of students with disabilities, the finding that athletic activities reduce the negative socio-emotional consequences of bullying, but non-athletic activities do not show a similar effect, once again demonstrates the importance of the type of activity (Bills, 2020; Peguero, 2008). Peer influence can also significantly impact motivation and the likelihood of participating in extracurricular activities, with both positive and negative effects (Iddrisu et al., 2024; Oberle et al., 2019). Taken together, the literature does not indicate a simple, uniform link between extracurricular involvement and bullying-related outcomes. Instead, the direction and strength of this link seem to depend on at least four interconnected factors: the type of activity, whether the focus is on victimization or perpetration, the level of supervision, and the quality of the program, as well as the broader social norms of the school environment. Studies looking at structured, supervised, and prosocial forms of participation generally report protective effects, while those examining norm-incongruent participation, specific activity categories, or settings with weaker supervision tend to show more mixed or even risk-increasing patterns (Berger et al., 2021; Hong et al., 2023; Peguero, 2008; Sheridan et al., 2022; Vandell et al., 2022). This pattern also aligns with broader reviews suggesting that extracurricular participation is not inherently protective on its own, but interacts with contextual factors and subgroup characteristics to influence bullying victimization and related school behaviors (Bills, 2021; Liu et al., 2024).

This point is especially important for the present study because our focus is not on students’ individual participation profiles but on the school-level availability of extracurricular opportunities. These are different constructs analytically. Individual participation may expose students to peer status competition, gender norm violations, or specific victimization risks, while the availability of extracurricular activities at the school level can also serve as an indicator of institutional capacity, organizational richness, adult supervision, and a broader environment of student engagement. In other words, a school that can offer a wider range of extracurricular opportunities may differ systematically from a school that cannot, not only in the provision of activities but also in school climate, staffing, routines, and the social environments that regulate behavior. This distinction helps clarify why mixed student-level findings do not rule out a school-level expectation in the present study.

From a social-ecological perspective, the expectation tested here is not that every extracurricular activity is equally beneficial, but that schools with more extensive extracurricular options tend to create organized, supervised, and supportive environments. These environments are expected to have fewer instructional barriers, less disorder, and fewer problems with aggression and misconduct. This idea aligns with school climate research showing that safer, better-organized, and more responsive school environments are linked to lower levels of bullying and aggression over time (Dorio et al., 2020; Thapa et al., 2013; Wang et al., 2020). Although the broader research on extracurricular participation is mixed, this study predicts a negative association at the school level because extracurricular availability is viewed as part of a larger institutional ecology rather than simply the number of student activities. Thus, the complex nature of the influence of extracurricular activity participation on peer bullying and aggressive behaviors requires consideration of several variables such as the type of activity, duration and intensity of participation, individual characteristics, and environmental factors (Berger et al., 2021; Peguero, 2008; Riese et al., 2015; Sheridan et al., 2022; Vandell et al., 2022).

### 1.1 A social-ecological perspective on school-level determinants of peer bullying

School-level aggressive and misconduct behaviors are increasingly understood as the result of complex interactions between individual traits and broader social and institutional contexts rather than as isolated personal issues (Hong and Espelage, 2012; Hymel and Swearer, 2015). In school psychology, bullying is widely recognized as a contextualized psychological behavior shaped by the social ecology in which students operate. The Social-Ecological Model, initially developed by Bronfenbrenner (1979), provides a well-established framework for examining such behaviors by emphasizing multiple, nested levels of influence, including school climate, social norms, adult supervision, and institutional resources. From a social-ecological perspective, schools serve as crucial developmental settings in which peer relationships, authority structures, and normative expectations converge (Espelage, 2014). School features such as supportive learning environments, consistent adult supervision, inclusive practices, and equitable organizational policies greatly influence the prevalence of bullying and aggressive behaviors (Thapa et al., 2013; Wang et al., 2020). In this way, bullying reflects not only individual interactions but also the psychosocial conditions created by the school environment.

Extracurricular activities play an important role in this social environment. When these activities are well-structured, inclusive, and properly supervised, they can promote prosocial norms, improve social connections, and increase students’ sense of belonging (Mahoney et al., 2005; Fredricks and Eccles, 2006). Conversely, participation in extracurricular activities held in settings with instructional barriers, weak institutional support, or limited supervision may fail to provide these protective psychological benefits and could even increase exposure to peer conflicts (Vandell et al., 2022). Therefore, the connection between extracurricular participation and bullying likely depends on broader school-level factors that influence students’ daily social interactions. Building on this idea, the present study adopts a social-ecological approach to examine how involvement in extracurricular activities relates to peer bullying and aggressive behaviors, with school-level contextual factors mediating this relationship. By including indicators such as issues that hinder instructional capacity (PHICS) and problems that interfere with students’ learning (PHSL), the study identifies structural features of the school’s psychosocial environment that may indirectly influence student behavior. More specifically, these school-level conditions are believed to operate through students’ psychological processes, such as their sense of belonging, perceived safety, and emotional regulation within the school setting. This perspective considers bullying as a core concern of school psychology rooted in institutional contexts rather than as a standalone individual issue. This study is based on Person-Environment Fit Theory (Eccles and Roeser, 2011) and Social Cognitive Theory (Bandura, 1986) to establish a strong theoretical foundation for these interactions. From the Person-Environment Fit perspective, bullying may emerge as a maladaptive response to a “stage-environment mismatch,” where structural factors, especially instructional capacity, fall short of meeting students’ developmental needs for stability. Additionally, Social Cognitive Theory emphasizes that the success of extracurricular activities in bullying prevention depends on a school environment that is organized enough to model and reinforce prosocial behaviors (Swearer et al., 2014).

Additionally, a recent meta-analytic review synthesizing studies from 2007 to 2023 reported a significant negative link between physical activity and bullying victimization, while finding no significant connection between physical activity and bullying perpetration (Liu et al., 2024). These findings highlight the importance of considering contextual and ecological factors when examining the effects of extracurricular participation. At the same time, they emphasize the importance of clearly distinguishing between individual participation, specific types of bullying involvement, and the broader institutional settings in which extracurricular activities are offered. In the present study, this distinction is key: the goal is not that all forms of participation reduce all types of bullying, but that schools with more available extracurricular activities, viewed within a social-ecological framework, will generally show fewer instructional barriers and lower levels of aggression and misconduct. To fully understand the complex relationship between extracurricular activities and peer bullying and aggressive behaviors, it is essential to go beyond activity-related and individual factors and include broader school-level conditions. In this context, the present study aims to offer a deeper and more comprehensive understanding of these relationships by explicitly considering PHICS and PHSL as contextual mediators within a school psychology and social-ecological framework.

### 1.2 Mediation an moderation role of PHICS and PHSL

From a school psychology perspective, instructional barriers and learning-related obstacles are not merely organizational challenges but also critical contextual stressors that shape students’ emotional security, sense of belonging, and behavioral regulation within the school environment (Thapa et al., 2013; Wang et al., 2020). Barriers to learning that arise in schools encompass multifaceted issues that directly affect the experiences of students and teachers (Zickafoose et al., 2024). These barriers include funding constraints and infrastructure deficiencies (Asmare et al., 2024; Mokgwathi et al., 2023). High student-teacher ratios (UNESCO, 2016; UNICEF, 2021) and a lack of technical capacity in resource management also negatively affect school-level utilization (Asmare et al., 2024; Zickafoose et al., 2024). Teachers’ competence and capability are also significant barriers to learning in schools (Asmare et al., 2024). A lack of professional development activities for teachers is one of the most significant barriers affecting the learning environment in schools (Gindo et al., 2020; Idika, 2021; Mokgwathi et al., 2023). Limited access to technology and challenging working conditions degrade teaching quality (Wang and Wang, 2023; Zickafoose et al., 2024), while intense workload (Kingdon et al., 2014; Salley and Shaw, 2015) and restricted participation in decision-making processes (Stosich, 2023) hinder effective teaching. Additionally, teachers’ frequent use of classical teaching methods and techniques, which rely on one-way transmission, negatively impacts students’ active participation in the learning process (Bentsi-Enchill, 2024; Idika, 2021). Inflated grades and grade inflation are significant school-based barriers that arise for reasons such as concealing failure, enhancing student motivation (Kızıltaş, 2024), and compromising the quality of education, leading to the employment of unqualified individuals and injustice (Arrafii, 2020; Chowdhury, 2018; Kızıltaş, 2024). Teachers’ difficulties with effective time management during lesson planning and curriculum implementation are another cause of instructional barriers (Asmare et al., 2024; Pabriaga, 2025). Lack of teaching materials and basic facilities is another common problem (Asmare et al., 2024; Gindo et al., 2020; Mokgwathi et al., 2023). Negative attitudes towards the learning process and low motivation are among the top student-centered barriers to learning (Bentsi-Enchill, 2024; Idika, 2021). Limited cooperation between parents, the local community, and the school has an indirect negative impact on the learning process (Gindo et al., 2020; Bentsi-Enchill, 2024). All these barriers are interconnected and prevent any education system from reaching its full potential (Zickafoose et al., 2024).

Peer bullying is a multidimensional problem that negatively affects students’ psychological, social, and academic development. This phenomenon is closely related not only to individual characteristics but also to structural and systemic factors in the school environment. Factors such as exclusion from educational environments, insufficient social support, physical infrastructure problems, and unfairness in assessment systems can increase the risk of students being exposed to bullying (Berchiatti et al., 2022; Juvonen et al., 2012). Especially, students with disabilities often face exclusion and inadequate support in the education system. This situation causes them to withdraw from social interactions and become more vulnerable to peer bullying (Rose et al., 2011). UNESCO (2017) emphasizes that inclusive education should include not only physical access but also social inclusion. Ainscow (2020) states that the lack of support for students with disabilities in the school environment leads them to experience social isolation and become more vulnerable to adverse experiences such as bullying.

Teacher support plays a decisive role in students’ ability to cope with bullying. However, insufficient teacher support increases students’ feelings of loneliness and makes them more vulnerable to negative peer interactions (Berchiatti et al., 2022; Wang and Chen, 2024). Berchiatti et al. (2022) found that the quality of student-teacher relationships is a determinant of students’ social status within peer groups and their exposure to bullying. A study conducted in China (Wang and Chen, 2024) found that teacher support mitigated the negative impact of school bullying on students’ sense of belonging. Atış-Akyol et al. (2018) state that teachers’ coping strategies for bullying are limited and often lack systematic intervention. Lack of social support weakens children’s ability to cope with stress and increases their tendency towards risky behaviors. On the other hand, crowded classrooms and inadequate physical infrastructure make it difficult for teachers to address students one-on-one, and social conflicts may be overlooked. This situation increases tension among students and paves the way for the spread of bullying behaviors. The inadequacy of the physical environment leads students to spend more time in unsupervised areas, exposing them to bullying (Atış-Akyol et al., 2018). Increasing grade inflation also leads to a sense of injustice among students. This situation has the potential to trigger negative emotions such as jealousy, competition, and distrust in social relations by blurring the distinction between working and non-working students. A lack of transparency in assessment processes can reinforce students’ perceptions of discrimination. UNESCO (2017) emphasizes that the lack of justice in education negatively affects relationships among students, which can foster bullying behavior. Diminished sense of belonging to school is both a precursor and a consequence of bullying (Wang and Chen, 2024). Juvonen et al. (2012) stated that peer relationships are determinant on students’ school belonging and academic participation and that exclusion is directly related to bullying.

Recent PISA-based bullying research has mainly concentrated on student-level victimization and its associated factors, such as grade repetition (Lian et al., 2021), subjective well-being (Katsantonis et al., 2024), school belonging, and changes across PISA cycles (Li et al., 2025), as well as the roles of teacher support, student characteristics, and school climate (Zhu and Teng, 2022). The current study broadens this focus by shifting the unit of analysis from students to schools and by utilizing the PISA 2022 School Questionnaire to investigate the structural relationships among the availability of extracurricular opportunities, instructional barriers, learning-related obstacles, and school-level aggressive and misconduct behaviors. In this way, the study’s novelty not only lies in using a more recent PISA cycle but also in conceptualizing extracurricular availability as a feature of school ecology rather than an individual participation factor, and in testing this idea within a moderated mediation framework.

### 1.3 Current study

Grounded in a social-ecological and school psychology framework, this study investigates the influence of school-level contextual factors on the relationship between the availability of extracurricular activities and school-level aggressive and misconduct behaviors, including peer intimidation. Specifically, the study tests a moderated mediation model in which problems that impair the school’s instructional capacity (PHICS) mediate this relationship, and factors that hinder students’ learning (PHSL) moderate its direct effect across countries participating in PISA. Although the wider research on extracurricular participation and bullying-related outcomes shows mixed results (Berger et al., 2021; Peguero, 2008; Riese et al., 2015; Sheridan et al., 2022), much of that variation seems to come from differences in activity type, participation level, whether the focus is on victimization or perpetration, and the specific social settings where participation happens. Additionally, many previous studies examine students’ individual participation patterns (Fredricks and Eccles, 2006; Mahoney et al., 2003; Peguero, 2008), whereas this study examines the availability of extracurricular opportunities across entire schools. This distinction is important from a theoretical perspective. At the school level, greater availability of extracurricular activities might not only mean more options for students but also reflect stronger organizational capacity, better adult supervision, more structured routines, and a more positive school climate. Therefore, this study predicts that schools with broader extracurricular offerings are likely to have lower levels of aggression and misconduct-related problems.

Research in school psychology consistently shows that school climate, including norms, adult supervision, and fairness of organizational practices, plays a key role in shaping bullying and aggressive behaviors (Thapa et al., 2013; Hymel and Swearer, 2015). In this framework, problems that impair the school’s PHICS can be viewed as a school-level contextual mechanism through which extracurricular activities are linked to school-level aggression and misconduct behaviors, while issues that PHSL can be understood as a contextual factor that influences the strength of the direct relationship between extracurricular activities and aggression- and misconduct-related school outcomes (Wang et al., 2020; Vandell et al., 2022).

Accordingly, the negative direction of H1 is not based on the assumption that all extracurricular activities are uniformly beneficial in all circumstances. Rather, it is derived from the expectation that, at the school level, the availability of extracurricular opportunities reflects a broader institutional ecology characterized by greater structure, supervision, engagement, and organizational support. In such school environments, instructional barriers are expected to be less severe, and aggression- and misconduct-related school problems are expected to occur at lower levels. By integrating these variables into a single moderated mediation model, the current study conceptualizes school-level aggressive and misconduct behaviors, including peer intimidation-related behaviors, as school-embedded psychological outcomes influenced by institutional conditions rather than as isolated individual behaviors. This approach also allows the study to move beyond a descriptive summary of mixed prior findings and to test a more specific theoretical claim: that extracurricular opportunities are most likely to be associated with lower aggression- and misconduct-related school outcomes when they are embedded in more functional instructional and learning environments. In this way, the study contributes to the school psychology literature with a cross-national perspective based on large-scale PISA data.

**Figure 1:**
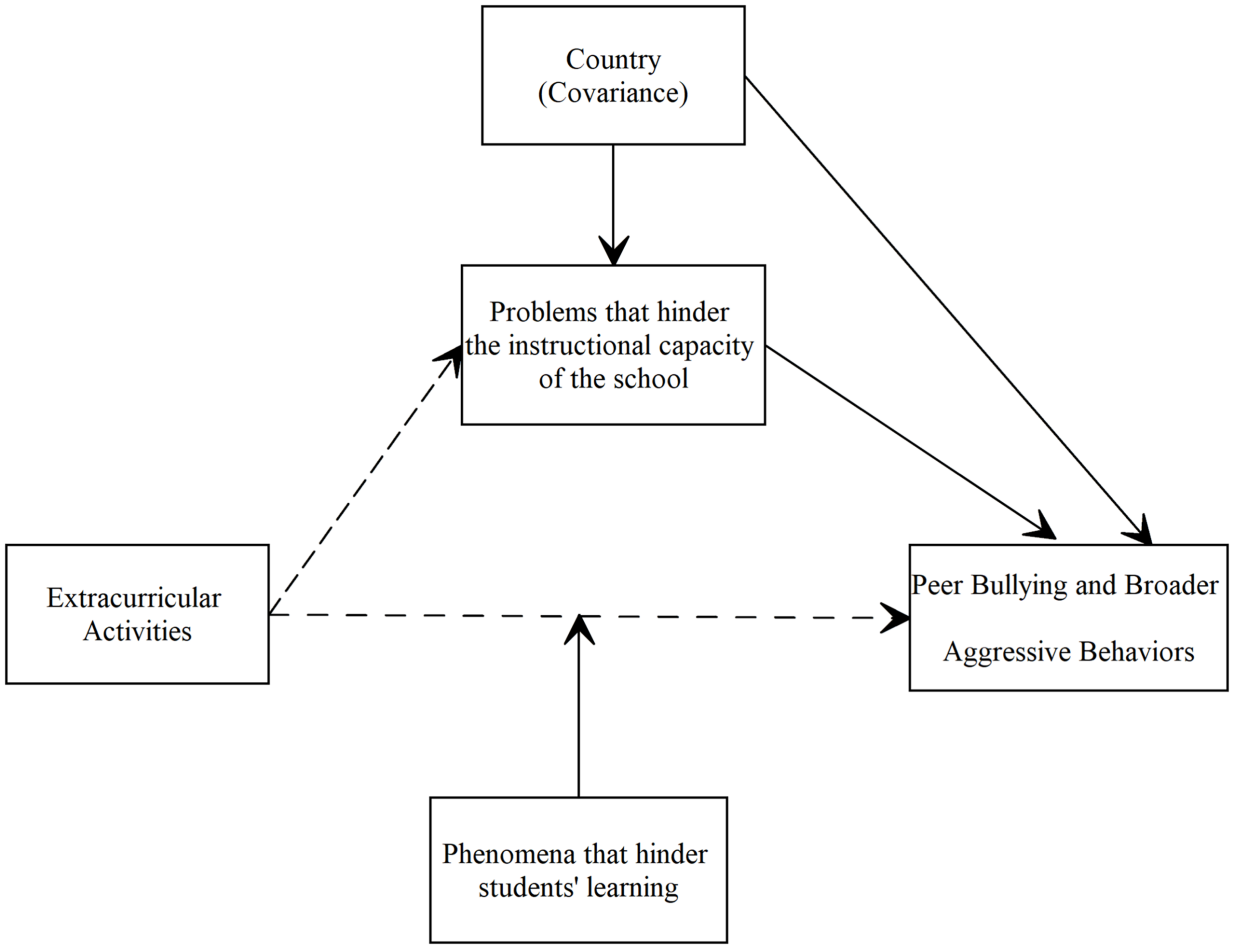
The hypothetical model (The dashed lines indicate a negative association in the opposite direction).

Figure 1 presents the hypothetical model illustrating the proposed relationships among the research variables within a school psychology and social–ecological framework.

In line with the hypothetical model presented in Figure 1 and the underlying social-ecological framework, and considering that the present study focuses on the school-level availability of extracurricular opportunities rather than individual participation patterns, the following directional hypotheses were formulated. Although previous findings have been mixed, the current study expects negative associations at the school level because broader extracurricular availability is viewed as an indicator of a more structured, supportive, and organizationally functional school environment.

- H1: Extracurricular activities are significantly and negatively associated with problems that hinder the instructional capacity of the school (PHICS).
- H2: Problems that hinder the instructional capacity of the school (PHICS) are significantly and positively associated with school-level aggressive and misconduct behaviors, including peer intimidation-related behaviors (PBABS).
- H3: Problems that hinder the instructional capacity of the school (PHICS) significantly mediate the association between extracurricular activities and PBABS.
- H4: Phenomena hindering students’ learning (PHSL) significantly moderate the direct association between extracurricular activities and PBABS. Specifically, the strength and direction of the association between extracurricular activities and PBABS vary by PHSL level.
- H5: Controlling for the effect of the Country covariate on both PHICS and PBABS, the overall model constitutes a moderated mediation framework, where PHICS mediates the association between extracurricular activities and PBABS, and PHSL moderates the direct path of this association.

## 2 Method

### 2.1 Research design

This study used a correlational research design based on secondary data analysis. Specifically, a cross-sectional design was employed, utilizing large-scale international assessment data from the PISA 2022 cycle, with schools as the primary unit of analysis. The correlational approach was considered suitable for examining the relationships among extracurricular activities, peer bullying, aggressive behaviors, and school-level contextual variables without implying causality. The focus on school-level variables reflects the theoretical assumption that peer bullying and aggressive behaviors are influenced by institutional and environmental conditions within the school context rather than solely by individual characteristics, consistent with a social–ecological perspective.

### 2.2 Population and sample

The study population consisted of schools that participated in the PISA 2022 assessment. The data were obtained from the publicly available PISA 2022 database provided by the OECD for research purposes (OECD, 2023). In accordance with the OECD’s official data policy, the use of these anonymized datasets for secondary analysis does not require special permission, provided that the source is properly cited.

The initial dataset included 21,629 schools from 80 participating countries, based on responses to the PISA 2022 School Questionnaire completed by school principals. These schools were chosen through PISA’s internationally standardized two-stage stratified sampling procedures to ensure national representation. However, although PISA uses a complex sampling design, the current study treated schools as independent units and did not apply sampling weights. This choice was based on the study’s goal: to examine structural relationships and theoretical mechanisms among variables rather than to estimate population parameters such as means or prevalence rates. In this context, unweighted analyses were considered more appropriate for the correlational, mediation, and moderation models tested in the study. The study focused on four main variables: (i) extracurricular activities, (ii) peer bullying and aggressive behaviors, (iii) problems hindering the school’s instructional capacity, and (iv) phenomena hindering students’ learning. Because response completeness varied across questionnaire items, the number of valid observations differed between descriptive and inferential analyses. Therefore, the initial sample of 21,629 schools should be understood as the full dataset available for the study, whereas the construction of the final analytic sample and the procedures for handling missing data are described in detail in the Data Analysis section.

### 2.3 Measures

#### 2.3.1 Extracurricular activities scale (EcAS)

Extracurricular activities were assessed using the Extracurricular Activities Scale (EcAS), which includes 10 dichotomous items (Q35) that measure the variety of extracurricular activities offered by the school during the academic year. Response options were “yes” and “no,” indicating whether each activity was available or not. In the original PISA dataset, “yes” responses were coded as 1 and “no” responses as 2. In this study, responses were recoded so that “yes” = 1 and “no” = 0. The total score was calculated by summing the affirmative responses, resulting in a range from 0 to 10. Higher scores reflect a greater availability of extracurricular activities at the school.

#### 2.3.2 Peer bullying and aggressive behaviors scale (PBABS)

Peer bullying and aggressive behaviors (PBABS) were assessed using Q27, part of the School Questionnaire for PISA 2022, which includes six Likert-type items, measuring the extent to which aggressive and misconduct-related behaviors occur at school. Items were rated on a four-point Likert scale ranging from 1 (not at all) to 4 (large extent). The scale measures a wide range of school-level problematic behaviors, including profanity, vandalism, theft, intimidation or verbal abuse among students, physical injuries caused by students to others, and intimidation or verbal abuse directed at teachers or non-teaching staff. Total scores ranged from 6 to 24, with higher scores indicating greater levels of school-level aggression and misconduct and lower scores reflecting a calmer, more orderly school environment. Although the scale includes an item directly addressing intimidation or verbal abuse among students, the overall PBABS reflects a broader school-level aggression and misconduct behavior construct rather than just bullying measure. The PBABS demonstrated excellent internal consistency (Cronbach’s alpha = 0.91). Accordingly, in this study, the outcome variable was understood to reflect school-level aggressive and misconduct behaviors, including peer intimidation-related actions, rather than just bullying in a narrow sense.

#### 2.3.3 School instruction hindrances scale (SIHS)

Problems that hinder the instructional capacity of the school (PHICS) were assessed using the School Instruction Hindrances Scale (SIHS). This scale includes 10 Likert-type items (Q25) designed to measure physical and human resource constraints that limit the school’s ability to meet its educational goals. Items were rated on a four-point scale from 1 (not at all) to 4 (A lot). Total scores range from 10 to 40, with higher scores reflecting greater instructional barriers. The SIHS demonstrated high internal consistency reliability (Cronbach’s alpha = 0.89). These indicators are viewed as contextual stressors within the school ecology that may indirectly affect students’ psychological experiences by impacting instructional quality and school climate.

#### 2.3.4 Students’ learning hindrances scale (SLHS)

Phenomena that hinder students’ learning (PHSL) were assessed using the Students’ Learning Hindrances Scale (SLHS). Although the original scale includes 11 Likert-type items (Q26), one item (“Students intimidating or bullying other students”) was excluded from the current analyses to prevent construct overlap with the dependent variable measuring peer bullying and aggressive behaviors. Consequently, the analyses were conducted using the remaining 10 items that measure conditions obstructing students’ learning processes. Items were rated on a four-point Likert scale from 1 (not at all) to 4 (A lot). Total scores ranged from 10 to 40, with higher scores indicating more frequent learning-related hindrances and lower scores reflecting a school environment that better supports learning. The modified SLHS showed excellent internal consistency reliability (Cronbach’s alpha = 0.90). The scale captures school-level learning environments that may undermine students’ emotional security, sense of belonging, and behavioral regulation.

#### 2.3.5 Reliability and validity

Internal consistency reliability for all study variables was assessed using Cronbach’s alpha. According to the criteria established by George and Mallery (2020), Cronbach’s alpha values above 0.70 are deemed acceptable, while those above 0.80 and 0.90 are considered good and excellent, respectively. All scales in the current study met or exceeded these thresholds, with Cronbach’s alpha coefficients ranging from 0.89 to 0.91. In addition to internal consistency, construct validity is supported by the OECD PISA 2022 technical documentation (OECD, 2023), which states that these school-level measures were created and validated for cross-national, large-scale comparative research.

### 2.4 Data analysis

All analyses were conducted using IBM SPSS Statistics and the PROCESS Macro. The data analysis proceeded in two stages: preliminary analyses and model testing. Prior to the analyses, response categories coded in the PISA database as “valid skip,” “not applicable,” “no response,” and “invalid” were treated as missing values. For each study variable, composite scores were created from the relevant questionnaire items after the recoding procedures described in the Measures section. Schools with missing responses on any item required to compute a given composite score were treated as missing for that scale score. For all multi-item scales, composite scores were calculated only for schools with valid responses on all items comprising the relevant scale. If a school had missing data on any item within a scale, the composite score for that scale was considered missing. Therefore, scale scores were based on complete item-level data for each construct.

In the preliminary stage, descriptive statistics were calculated to summarize the study variables and examine the data distributions. For the extracurricular activity items, frequencies and percentages were reported. For the Likert-type items and composite variables, means, standard deviations, skewness, and kurtosis values were computed. Prior to defining the final sample, five countries (Argentina, Germany, Japan, Singapore, and Spain) were excluded from the original 80 country dataset because they had completely missing data on the outcome variable (PBABS). Descriptive statistics were calculated based on the final analytic sample of 16,555 schools, which was obtained after applying listwise deletion for missing data. To ensure comparability across the main inferential analyses, a harmonized complete-case dataset was constructed for all variables included in the primary model. Specifically, schools with missing data on any of the variables required for the main analyses were excluded through listwise deletion. The final analytic sample, therefore, consisted of 16,555 schools across the remaining 75 countries with complete data on extracurricular activities (EcAS), problems hindering the school’s instructional capacity (PHICS), phenomena hindering students’ learning (PHSL), peer bullying and aggressive behaviors (PBABS), and Country. Pearson correlation analyses, multiple regression analyses, and the conditional process analyses were conducted using this same analytic sample.

In the second stage, the hypothesized model was tested using the PROCESS Macro for SPSS (Model 5; Hayes, 2018). In this model, PHICS was specified as the mediator of the association between extracurricular activities and peer bullying and aggressive behaviors, whereas PHSL was specified as a moderator of the direct association between extracurricular activities and peer bullying and aggressive behaviors. To facilitate the interpretation of the moderation effects and minimize potential multicollinearity, the moderator variable (PHSL) was mean-centered prior to constructing the interaction term (Aiken and West, 1991). Furthermore, to rigorously account for the nested nature of the cross-national data and control for within-country dependence, Country Fixed Effects were implemented. Specifically, 74 country dummy variables were created and included as covariates in the model to control for unobserved country-level heterogeneity. The statistical significance of the indirect effect and the conditional direct effects was evaluated using a bootstrapping procedure with 5,000 resamples and 95% bias-corrected confidence intervals. An effect was considered statistically significant when the confidence interval did not include zero. Finally, to comprehensively probe the significant interaction and identify the exact regions where the conditional direct effect of extracurricular activities on PBABS differs statistically from zero, the Johnson-Neyman technique was used (Hayes, 2018).

## 3 Results

### 3.1 Preliminary analysis

Table 1 presents descriptive statistics regarding the availability of extracurricular activities across schools, based on responses to the item, “Which of the following activities does your school offer to students?”.

**Table 1:** Descriptive statistics on extracurricular activities offered by schools to students (*N* = 16,555).

Item	Extracurricular activity	Yes *f* (%)	No *f* (%)
1	Band, orchestra, or choir	8,019 (48.4)	8,536 (51.6)
2	School play or school musical	7,849 (47.4)	8,706 (52.6)
3	School yearbook, newspaper, or magazine	7,664 (46.3)	8,891 (53.7)
4	Volunteering or service activities	11,994 (72.4)	4,561 (27.6)
5	Mathematics club	6,370 (38.5)	10,185 (61.5)
6	Mathematics competitions	11,493 (69.4)	5,062 (30.6)
7	Chess club	5,658 (34.2)	10,897 (65.8)
8	Club with a focus on computers (e.g., programming or coding)	7,486 (45.2)	9,069 (54.8)
9	Art club or art activities	10,264 (62.0)	6,291 (38.0)
10	Sporting team or sporting activities	14,395 (87.0)	2,160 (13.0)

As shown in Table 1, sporting activities (87.0%) and volunteering or service activities (72.4%) were the most commonly offered extracurricular options, followed closely by mathematics competitions (69.4%). In contrast, activities such as chess clubs (34.2%), mathematics clubs (38.5%), and computer-focused clubs (45.2%) were less prevalent. Overall, the findings indicate significant variation in the range of extracurricular opportunities available to students across different schools.

Table 2 presents descriptive statistics regarding the frequency of aggressive behaviors exhibited by students at school, based on responses to the item, “To what extent is each of the following behaviors a problem in your school?”.

**Table 2:** Descriptive statistics on the frequency of aggressive behaviors exhibited by students at school (*N* = 16,555).

Item	Aggressive behavior	Mean	*SD*
1	Profanity	1.97	0.87
2	Vandalism	1.71	0.84
3	Theft	1.52	0.76
4	Intimidation or verbal abuse among students (including texting, emailing, etc.)	1.96	0.82
5	Physical injury caused by students to other students	1.55	0.76
6	Intimidation or verbal abuse of teachers or non-teaching staff	1.48	0.76
	Total	1.70	0.67

SD = standard deviation. Responses were measured on a 4-point Likert scale (1 = Not at all, 4 = Large extent)”.

As shown in Table 2, the overall PBABS score indicated generally low but non-negligible levels of school-level aggression and misconduct (*M* = 1.70, *SD* = 0.67). Among individual items, principals most often reported profanity (*M* = 1.97, *SD* = 0.87) and intimidation or verbal abuse among students (*M* = 1.96, *SD* = 0.82). In contrast, intimidation or verbal abuse directed at teachers or non-teaching staff was reported the least frequently (*M* = 1.48, *SD* = 0.76). These findings suggest that the outcome variable reflects a broader pattern of school-level aggressive and misconduct behaviors rather than a narrow bullying-only construct.

Table 3 presents descriptive statistics regarding the problems that hinder the instructional capacity of schools, based on principals’ responses to the item, ‘Is your school’s capacity to provide instructional hindered by any of the following issues?’

**Table 3:** Descriptive statistics on problems hindering the instructional capacity of schools (*N* = 16,555).

Item	Instructional hindrance	Mean	*SD*
1	A lack of teaching staff	2.15	1.03
2	Inadequate or poorly qualified teaching staff	1.84	0.89
3	A lack of assisting staff	2.06	1.04
4	Inadequate or poorly qualified assisting staff	1.71	0.91
5	A lack of educational material (e.g., textbooks, IT equipment, library or laboratory material)	2.00	1.04
6	Inadequate or poor quality educational material	1.95	0.99
7	A lack of physical infrastructure (e.g., building, grounds, heating/cooling, lighting and acoustic systems)	1.99	1.08
8	Inadequate or poor quality physical infrastructure	1.97	1.05
9	A lack of digital resources (e.g., desktop or laptop computers, internet access, learning management systems)	2.08	1.08
10	Inadequate or poor quality digital resources	2.08	1.06
	Total	1.98	0.72

SD = standard deviation. Responses were measured on a 4-point Likert scale (1 = not at all, 4 = A lot).

As shown in Table 3, the PHICS overall mean score indicated a small level of instructional barriers across schools (M = 1.98, SD = 0.72). Among specific issues, principals identified a lack of teaching staff (*M* = 2.15, *SD* = 1.03) and assisting staff (*M* = 2.06, *SD* = 1.04) as the most prominent staffing challenges. Conversely, concerns about inadequate or poorly qualified teaching staff scored lower (*M* = 1.84, *SD* = 0.89). This pattern suggests that perceptions of staffing quantity pose a more immediate instructional challenge than perceptions of staff quality at the school level.

Table 4 presents descriptive statistics regarding phenomena that hinder students’ learning, based on principals’ responses to the item, “In your school, to what extent is the learning of students hindered by the following phenomena?”.

**Table 4:** Descriptive statistics on phenomena hindering students’ learning (*N* = 16,555).

Dimension	Item	Learning hindrance	Mean	*SD*
Hindrances caused by the teacher	6	Teachers not meeting individual students’ needs	2.00	0.80
	7	Teacher absenteeism	1.90	0.88
	8	Staff resisting change	1.95	0.88
	9	Teachers being too strict with students	1.78	0.75
	10	Teachers not being well prepared for classes	1.78	0.81
		Dimension total	1.88	0.66
Hindrances caused by the student	1	Student truancy	2.36	0.91
	2	Student skipping classes	2.25	0.87
	3	Students lacking respect for teachers	2.01	0.83
	4	Student use of alcohol or illegal drugs	1.54	0.80
	11	The student not being attentive	2.55	0.81
		Dimension total	2.14	0.66
		Total (PHSL)	2.01	0.60

SD = standard deviation. PHSL = Phenomena Hindering Students’ Learning. Responses were measured on a 4-point Likert scale (1 = Not at all, 4 = A lot). Item 5 (“Students intimidating or bullying other students”) was excluded from the analysis to prevent construct overlap with the outcome variable.

As shown in Table 4, the overall PHSL score indicated a small level of learning obstacles across schools (M = 2.01, SD = 0.60). Among the specific phenomena, principals most frequently reported students’ lack of attention (*M* = 2.55, *SD* = 0.81) and student truancy (*M* = 2.36, *SD* = 0.91) as major hindrances. In contrast, student use of alcohol or illegal drugs was reported least frequently (*M* = 1.54, *SD* = 0.80). These findings suggest that learning-related obstacles are primarily associated with student motivation and daily school engagement rather than extreme behavioral violations.

As shown in Table 5, the skewness and kurtosis coefficients for the investigated variables were generally between ± 2 (absolute skewness and kurtosis), indicating that the data were normally distributed (Kline, 2016). Correlation analysis showed that all variables were significantly related to one another at the *p* < 0.01 level. PBABS was positively associated with PHSL and PHICS. Conversely, EcAS was negatively correlated with PBABS, PHICS, and PHSL. A moderate positive relationship was observed between PHICS and PHSL. Taken together, these correlations indicate that greater extracurricular availability is associated with lower levels of broader school-level aggressive and misconduct behaviors, whereas instructional and learning-related barriers are associated with higher levels of such school problems.

**Table 5:** Descriptive statistics and inter-correlations among study variables (*N* = 16,555).

Variables	*M*	*SD*	Skewness	Kurtosis	(1)	(2)	(3)	(4)
1. EcAS	5.508	2.598	−0.117	−0.763	--			
2. PHSL	20.118	5.978	0.712	0.643	−0.089	--		
3. PHICS	19.830	7.198	0.555	−0.399	−0.122	0.443	--	
4. PBABS	10.199	4.046	1.510	2.548	−0.053	0.537	0.263	--

M = mean; SD = standard deviation. EcAS = Extracurricular Activities Scale; PHSL = Phenomena Hindering Students’ Learning; PHICS = Problems Hindering the Instructional Capacity of Schools; PBABS = Peer Bullying and Aggressive Behavior. All correlations are significant at the *p* < 0.01 level.

### 3.2 Moderated mediation analysis

Table 6 presents the regression results regarding the mediating role of PHICS and the moderating role of PHSL in the relationship between EcAS and PBABS. As shown in Table 6, in the first model, EcAS was negatively associated with PHICS (*B* = −0.361, *SE* = 0.024; 95% CI [−0.407, −0.315]), supporting hypothesis H1. Furthermore, this model explained 17.5% of the variance in PHICS (*R*^2^ = 0.175). In the model where PBABS was the outcome variable, PHICS was positively associated with PBABS (*B* = 0.020, *SE* = 0.004; 95% CI [0.012, 0.029]), and H2 was supported. These findings indicate that school-level aggressive and misconduct behaviors tend to be more prevalent in school contexts characterized by greater instructional barriers.

**Table 6:** Regression results for direct, moderated, and indirect effects on PBABS (*N* = 16,555).

Model/effect	*B*	*SE*	*t*	*p*	95% CI [LL, UL]
Model 1: PHICS (*R^2^* = 0.175)					
EcAS	−0.361	0.024	−15.290	0.001	[−0.407, −0.315]
Model 2: PBABS (*R*^2^ = 0.387)					
EcAS	0.008	0.012	0.713	0.476	[−0.014, 0.031]
PHICS	0.020	0.004	4.785	0.001	[0.012, 0.029]
PHSL	0.350	0.005	69.808	0.001	[0.340, 0.360]
EcAS × PHSL (*ΔR^2^* = 0.0002)	−0.004	0.002	−2.428	0.015	[−0.007, −0.001]

Indirect effect	Effect	Boot *SE*			95% Boot CI [LL, UL]
EcAS→ PHICS → PBABS	−0.007	0.002			[−0.011, −0.004]

B: unstandardized regression coefficient; SE: standard error; CI: confidence interval; LL: lower limit; UL: upper limit; *R*^2^: coefficient of determination; Δ*R*^2^: *R*^2^ change due to interaction; df: degrees of freedom. EcAS: Extracurricular Activities Scale; PHICS: Problems Hindering the Instructional Capacity of Schools; PHSL: Phenomena Hindering Students’ Learning; PBABS: Peer Bullying and Aggressive Behavior. The moderator variable (PHSL) was mean-centered prior to the analysis to ease interpretation and avoid multicollinearity. To account for within-country dependence, Country Fixed Effects were included in the models using 74 country dummy variables; their coefficients are omitted from the table for brevity.

As shown in Table 6, the interaction term between EcAS and the mean-centered PHSL (EcAS × PHSL) demonstrated a statistically significant effect on PBABS (*B* = −0.004, *SE* = 0.002; 95% CI [−0.007, −0.001]). An examination of the *R*^2^ change associated with this interaction revealed that its contribution to the explained variance was Δ*R*^2^ = 0.0002. These findings indicate that PHSL significantly moderates the direct association between EcAS and PBABS, thereby supporting Hypothesis H4. To clarify this interaction and determine exactly where the conditional effect differs from zero, the Johnson-Neyman technique was applied. The analysis revealed that the direct effect of EcAS on PBABS is statistically significant only when the mean-centered PHSL score is below −4.759 (accounting for 22.89% of the sample) and above 17.933 (accounting for 0.92% of the sample).

The indirect effect of EcAS on PBABS through PHICS was estimated using 5,000 bootstrap samples. The analysis revealed a significant negative indirect effect (*B* = −0.007, *SE* = 0.002), and the 95% bootstrap CI did not contain zero ([−0.011, −0.004]). This finding indicates that PHICS has a significant mediating role in the relationship between EcAS and PBABS, thereby supporting Hypothesis H3. Furthermore, the overall model, including all independent, mediating, and moderating variables along with the Country Fixed Effects, explained 38.7% of the variance in PBABS (*R*^2^ = 0.387). By accounting for within-country dependence, the simultaneous significance of PHICS’s mediating role and PHSL’s moderating role on the direct path indicates that the overall model constitutes a moderated mediation framework, confirming Hypothesis H5. Accordingly, extracurricular activities were linked to lower levels of broader school-wide aggressive and misconduct behaviors indirectly by reducing instructional barriers, while the direct relationship depended on the school’s learning-related conditions.

Table 7 presents the conditional effects of EcAS depending on whether the level of mean-centered PHSL is at −1 *SD*, Mean, or +1 *SD*. Accordingly, at low levels of PHSL (−1 *SD*), the effect is positive and significant (*B* = 0.031, *SE* = 0.015, *t* = 2.132, *p* = 0.033; 95% CI [0.003, 0.060]). At the average level of PHSL (Mean), the effect is statistically non-significant (*B* = 0.008, *SE* = 0.012, *t* = 0.713, *p* = 0.476; 95% CI [−0.014, 0.031]). Similarly, at high levels of PHSL (+1 *SD*), the effect is negative but statistically non-significant (*B* = −0.015, *SE* = 0.015, *t* = −0.978, *p* = 0.328; 95% CI [−0.045, 0.015]). These conditional effects show that the relationship between extracurricular activities and broader school-level aggressive and misconduct behaviors is not uniform across school contexts but varies with the severity of learning-related hindrances.

**Table 7:** Conditional direct effects of EcAS on PBABS across levels of PHSL (*N* = 16,555).

PHSL condition	Level	*B*	*SE*	*t*	*p*	95% CI [LL, UL]
Low	−1 *SD* (−5.978)	0.031	0.015	2.132	0.033	[0.003, 0.060]
Average	Mean (0.000)	0.008	0.012	0.713	0.476	[−0.014, 0.031]
High	+1 *SD* (5.978)	−0.015	0.015	−0.978	0.328	[−0.045, 0.015]

SD: standard deviation; B: unstandardized coefficient; SE: standard error; CI: confidence interval; LL: lower limit; UL: upper limit. PHSL: Phenomena Hindering Students’ Learning; EcAS: Extracurricular Activities Scale; PBABS: Aggressive Behavior at School. The moderator variable (PHSL) was mean-centered. Based on the Johnson-Neyman technique, the conditional direct effect of EcAS on PBABS becomes statistically significant only when the mean-centered PHSL score is below −4.759 (accounting for 22.89% of the sample) and above 17.933 (accounting for 0.92% of the sample).

**Figure 2:**
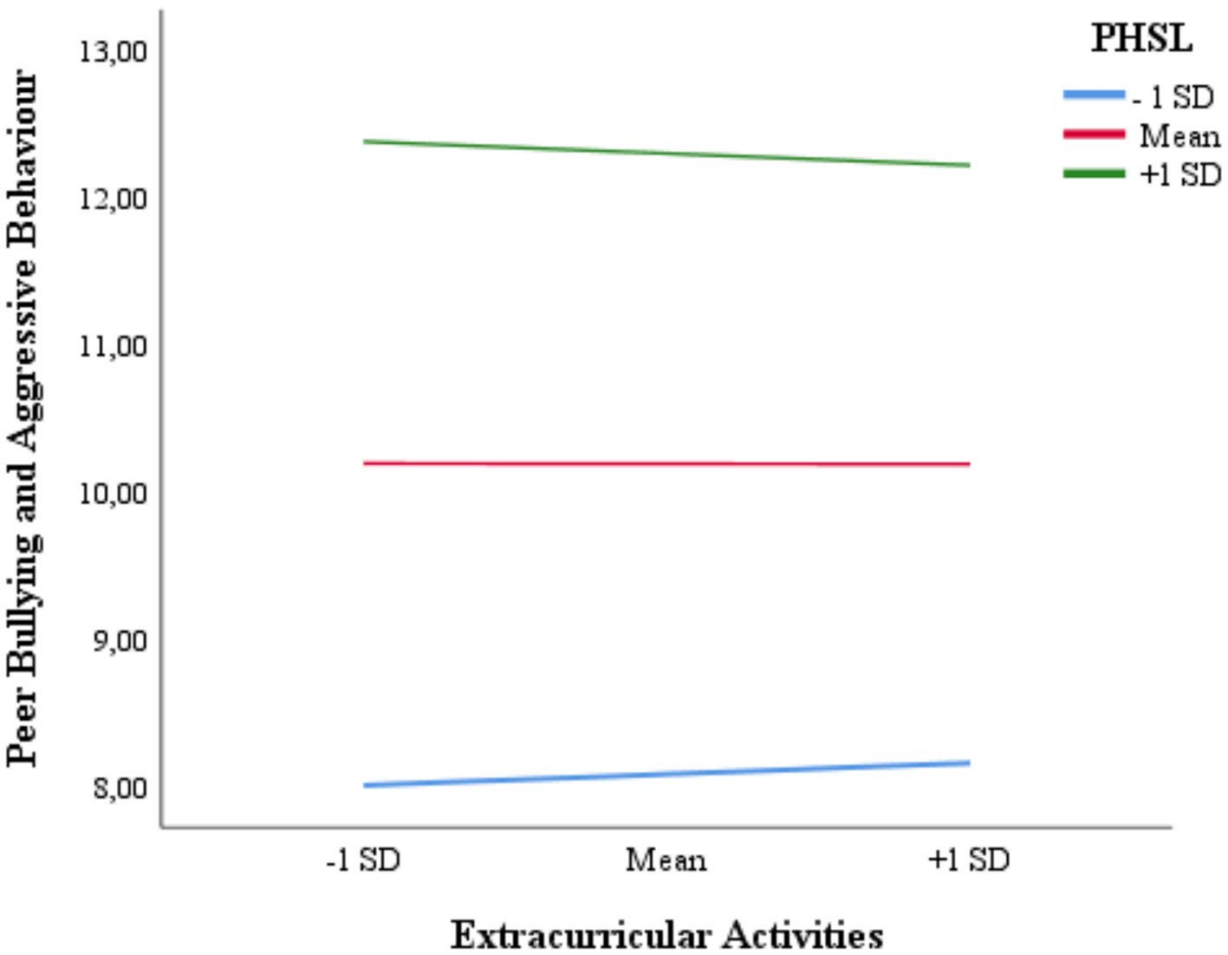
Moderating effect of phenomena hindering students’ learning (PHSL) on the relationship between extracurricular activities and peer bullying and aggressive behavior (low, mean, and high PHSL levels correspond to −1 *SD*, mean, and +1 *SD*, respectively).

The results of the simple slope analyses, conducted to examine how the conditional effects of EcAS on PBABS vary across mean-centered PHSL levels (Low, Mean, High), are visually represented in Figure 2. An examination of the conditional effects reveals that when the level of PHSL is high (+1 *SD*) or at the average (Mean), the relationship between the availability of EcAS and PBABS is not statistically significant. Conversely, when the PHSL level is low (−1 *SD*), a significant positive association emerges, indicating that in school environments with very few learning hindrances, greater availability of extracurricular activities is unexpectedly linked to slightly higher levels of aggressive and misconduct behaviors. Thus, Figure 2 further illustrates that extracurricular activities are related not simply to peer bullying in a narrow sense, but also to broader school-level aggressive and misconduct behaviors, whose patterns are highly context-dependent and do not uniformly serve a protective function across all learning environments.

## 4 Discussion

Before exploring the theoretical implications, it is helpful to briefly summarize the study’s main empirical findings. At the bivariate level, extracurricular activities were negatively associated with PBABS, though the direct effect was relatively small. When school-level contextual factors were included in the model, the average direct effect of extracurricular activities on PBABS became non-significant, while the indirect effect through PHICS remained significant. Additionally, PHSL showed a strong positive relationship with PBABS and significantly moderated the direct link between extracurricular activities and PBABS, suggesting that the relationship varied across levels of learning-related obstacles. Overall, the final moderated mediation model accounted for 38.7% of the variance in PBABS, suggesting that the connection between extracurricular opportunities and school-level aggressive and misconduct behaviors is better understood in the context of broader instructional and learning conditions rather than as a simple direct relationship.

This study examines school-level aggressive and misconduct behaviors, including peer bullying-related behaviors, not merely as individual deviations or a lack of activity, but as a contextual phenomenon rooted in the social-ecological perspective. By redefining these school-level behavior problems as outcomes embedded within the institutional, instructional, and relational fabric of the school’s “lifeworld,” situated within the layered structure of ecological systems theory, this research adds to the scholarly literature. The findings support school psychology approaches that view aggression, misconduct, and bullying-related school problems as relational issues influenced by broad institutional, instructional, and learning environments, rather than as solely individual problems. Within this social-ecological framework, which considers the individual as interconnected with micro- and macro-systems rather than independent, school-level aggression and misconduct are conceptualized as social and institutional outcomes. In this context, although results show that EcAS are generally linked to lower levels of school-level aggressive and misconduct behaviors, this protective connection is mediated by school-level contextual factors rather than a direct influence. In other words, the protective role of EcAS is better understood within the school’s broader ecological system of instructional and learning conditions than as a simple bullying-prevention effect. The findings are discussed below, organized by the direct relationships, the role of school-level mechanisms, and the underlying processes through which these links emerge.

### 4.1 The direct link between extracurricular activities and peer bullying

At the bivariate level, extracurricular activities were negatively associated with peer bullying and aggressive behavior, which is consistent with the strand of prior research suggesting that structured and supervised extracurricular opportunities may function as protective school resources, even though the broader literature remains mixed (Fredricks and Eccles, 2006; Mahoney et al., 2003; Riese et al., 2015). These findings support developmental theories suggesting that structured activities can enhance social skills, peer relationships, and prosocial norms (Berger et al., 2021; Mahoney et al., 2005). From a philosophical standpoint, the school is not just a place where knowledge is exchanged but a social institution where students build their own “domain of existence” within the interaction between environment and behavior highlighted by Bandura (1977). However, the strength of this direct relationship was relatively small. This necessitates caution regarding the practical significance of the findings. Although the results are statistically significant due to the large sample size, the small effect size suggests that extracurricular activities play a limited role in preventing bullying in isolation. More precisely, based on the operationalization of PBABS, this association should be understood as referring to broader school-level aggressive and misconduct behaviors, including peer intimidation-related behaviors, rather than to bullying alone. Furthermore, when both the mediator and moderator were included simultaneously in the model, the average direct effect of extracurricular activities on peer bullying and aggressive behavior became non-significant. This suggests that the direct relationship is better understood as context-dependent rather than consistent across schools. Specifically, the conditional effect of extracurricular activities varied by PHSL level, indicating that the direction and strength of the direct relationship changed depending on the extent to which students’ learning was hindered. This modeling challenges the simple idea that merely adding more extracurricular options can, on its own, significantly reduce bullying behavior. Therefore, the current findings should not be taken to mean that extracurricular activities are always protective in every context; instead, they indicate that, at the school level, increased availability of extracurricular activities is linked to fewer aggression- and misconduct-related problems under specific instructional and learning circumstances.

### 4.2 Mediating and moderating roles of school-level factors

The limitations of the direct link prompt consideration of school-level mechanisms, including both mediation and moderation (H2, H3, H4, H5). From a social-ecological perspective, this finding is critically important. Bronfenbrenner’s (1979) ecological theory highlights that interconnected systems, including institutional practices, norms, and resource availability, shape individual behaviors. At this stage, bullying appears as a harmful social mechanism used to fulfill the need for power and recognition that the institutional structure fails to provide (Olweus, 1993). Current findings indicate that extracurricular activities are part of the school microsystem. However, their psychological benefits depend on a larger mesosystem that includes instructional quality, organizational capacity, and the learning environment. When schools experience staffing shortages, limited resources, weak instructional support, or widespread learning obstacles, the potential of extracurricular activities to foster emotional security and social regulation may be significantly diminished (Thapa et al., 2013; Wang et al., 2020). Within this framework, the present findings suggest that problems hindering the school’s instructional capacity (PHICS) function as a mediating mechanism, whereas phenomena hindering students’ learning (PHSL) function as a moderating contextual condition that shapes the direct association between extracurricular activities and peer bullying and aggressive behaviors. At the same time, since the outcome variable reflects a broader pattern of school-level aggression and misconduct, these mechanisms should also be seen as helping to explain variation in wider school behavior problems rather than just bullying in a narrow sense.

The mediating role of problems that hinder instructional capacity (PHICS) highlights the institutional roots of bullying. A school environment lacking sufficient physical and human resources creates an ontological “void” in which adult supervision is inconsistent, and norms of respect and inclusion weaken. Schools with staffing shortages, poor infrastructure, and limited instructional support may unintentionally foster environments where supervision is unreliable, and norms of respect and inclusion diminish. Past research indicates that these structural issues are linked to a worse school climate and increased student misconduct (Espelage, 2014; Hymel and Swearer, 2015). In this way, PHICS can act as a distal stressor that is indirectly associated with a higher risk of bullying by relating to lower psychological safety and cohesion within the school. Theoretically, this phenomenon can be further elucidated through the Social Cognitive Theory (Bandura, 1986). According to this framework, specifically within the principle of reciprocal determinism, school-level educational barriers function as negative environmental determinants that may attenuate the protective effects of extracurricular participation (Swearer et al., 2014). From a Person-Environment Fit perspective (Eccles and Roeser, 2011), these educational deficiencies create a mismatch that stifles the sense of social belonging typically fostered by such activities, thereby positioning bullying behavior as a dysfunctional coping strategy in response to institutional inadequacies (Hymel and Swearer, 2015; Thapa et al., 2013). In this study, however, the implications of PHICS go beyond just bullying and more broadly relate to school climates where aggressive and misconduct-related issues are more likely.

At the same time, the role of PHSL should be interpreted differently in the current model. Instead of acting as a second mediator, PHSL seems to function as a moderator of the direct link between extracurricular activities and peer bullying and aggressive behaviors. However, it is essential to critically assess the practical importance of this moderation. Although the interaction between extracurricular activities and PHSL is statistically significant, the very small change in explained variance (ΔR2 = 0.0002) suggests that its real-world impact is quite limited. The statistical significance of this crossover effect is mostly due to the large sample size (*N* = 16,555). An examination of the conditional effects indicates that the direct protective relationship between extracurricular activities and aggressive behaviors is largely non-significant for the vast majority of school environments. Instead, a significant direct association emerges primarily in schools with very low learning barriers, where greater availability of activities is unexpectedly linked to slightly higher levels of aggression. Nonetheless, the overall size of this conditioned interaction remains modest. From a social-ecological perspective, this moderating pattern supports the idea that extracurricular activities do not deliver their benefits in isolation but within learning environments that can either enhance or diminish their impact on bullying-related outcomes (Thapa et al., 2013; Wang et al., 2020). In this way, PHSL serves as a contextual factor that influences how extracurricular opportunities relate to peer bullying and aggressive behaviors, thereby supporting a moderated mediation model rather than a simple mediation explanation. This interpretation also aligns with the theoretical rationale of the present study, which suggests that mixed findings in the broader literature are likely to become more consistent once extracurricular opportunities are viewed as school-level contextual resources rather than as undifferentiated forms of individual participation.

### 4.3 Understanding the mechanism: why context matters more than activities

Importantly, the findings of the present study suggest that extracurricular activities are associated with peer bullying and aggressive behaviors through a context-dependent mechanism rather than a straightforward one pathway. Extracurricular programs can help foster a more positive learning climate by increasing student engagement, strengthening school attachment, and reinforcing norms of cooperation and mutual respect (Feldman et al., 2021; Martinez et al., 2016). However, as Dewey (1916) emphasizes, the success of these social activities is closely linked to the school’s overall democratic and inclusive atmosphere. When learning environments face ongoing challenges, such as widespread disengagement or perceptions of unfairness, these benefits might not fully materialize. In such cases, extracurricular participation could even worsen inequalities by benefitting students who are already engaged while leaving marginalized groups vulnerable to exclusion or victimization (Berger et al., 2021; Peguero, 2008).

The current results show that extracurricular activities are indirectly linked to lower bullying-related outcomes through issues that hinder the school’s instructional capacity (PHICS), while the direct relationship between extracurricular activities and peer bullying and aggressive behaviors varies depending on the level of problems affecting students’ learning (PHSL). Therefore, the findings align more with a moderated mediation model rather than a simple direct effect or parallel mediation approach. Importantly, this pattern should be understood in the context of the present study’s school-level design. Unlike studies that focus on individual participation, the current analysis considers extracurricular availability as a contextual school resource. From this perspective, the observed negative association does not contradict the mixed literature; instead, it clarifies the conditions under which more negative or more protective patterns are likely to occur. Because the dependent variable reflects a broader school-level aggression and misconduct construct, these results are best interpreted as indicating that extracurricular activities may help reduce aggression- and misconduct-related problems at school, including issues with peers bullying-related behaviors, when embedded in more supportive organizational contexts. When school learning environments face serious challenges, the potential psychosocial benefits of extracurricular activities may be weakened, reshaped, or become contingent on contextual supports that are often unequally distributed across schools.

The findings also call for a philosophical shift in school behavior prevention and intervention strategies. Instead of viewing bullying solely as a behavior to be controlled through specific interventions, the results support understanding it as a symptom of deeper institutional issues. From this perspective, bullying reflects how students experience power, belonging, and recognition within the school as a social institution. Schools that do not meet students’ instructional and learning needs may inadvertently foster environments in which aggression becomes a normal form of social interaction (Hymel and Swearer, 2015; Wang et al., 2020). Accordingly, the policy implications of the present study extend beyond bullying prevention in a narrow sense and point toward broader efforts to improve school climate and manage behavior. Overall, this study contributes to the literature by empirically showing that extracurricular activities are most effective as protective tools when embedded within supportive instructional and learning settings. The findings support calls for comprehensive, school-wide strategies to reduce aggression- and misconduct-related school problems by combining extracurricular programs with investments in teaching capacity, inclusive teaching practices, and learning environments that foster psychological safety and a sense of belonging (Hymel and Swearer, 2015; Thapa et al., 2013). By placing extracurricular activities within a broader social-ecological framework, this study helps develop a clearer understanding of how institutional conditions influence student behavior across different educational systems.

### 4.4 Limitations and future research

Despite its strengths, including a large cross-national sample and a theoretically grounded moderated mediation model, the present study has several limitations that should be acknowledged. First, the use of cross-sectional data restricts the ability to establish causal relationships and temporal ordering among extracurricular activities, school-level barriers, and peer bullying and aggressive behaviors. Therefore, the findings should be interpreted as statistical associations rather than causal effects. Although the proposed model is theoretically based and consistent with social-ecological frameworks (Bronfenbrenner, 1979; Hymel and Swearer, 2015), longitudinal studies are necessary to observe how changes in instructional capacity, learning environments, and extracurricular offerings develop over time and affect bullying patterns.

Second, the study relied exclusively on school principals’ reports to assess bullying-related behaviors and school-level barriers. This reliance introduces the potential for single-informant bias and common method variance. While principal reports offer a valuable institutional perspective, they may fail to fully capture the frequency and complexity of bullying incidents experienced by students, or they may underreport such events due to social desirability concerns. Consequently, the current findings may be limited in their ability to accurately reflect the prevalence and relational nuances of bullying. Future research would benefit from validating these results through multi-informant designs that triangulate data from multiple sources, such as student and teacher reports, to better capture the psychological and relational aspects of bullying and to strengthen the credibility of the interpretation.

Third, although the large sample size enabled robust statistical testing, the observed effect sizes were relatively small. Therefore, statistical significance should not be equated with strong practical impact. These coefficients warrant a cautious interpretation, as they reflect consistent trends across the extensive sample but may hold limited explanatory power for individual schools. While such effects are common and meaningful in large-scale educational research, future studies could explore whether these relationships vary across cultural contexts, educational systems, or school types. Cross-level and multilevel modeling approaches may help clarify how national policies, school norms, and classroom practices collectively influence bullying-related outcomes (Thapa et al., 2013; Espelage, 2014).

Fourth, the operationalization of extracurricular activities focused on their availability, without measuring participation rates, intensity, or inclusiveness. This limits the substantive interpretation of what “extracurricular activities” actually represent in practice. Therefore, the findings should not be overgeneralized to imply that the mere existence of these activities is protective, as the quality and context of participation are crucial for positive outcomes. Prior research indicates that not all extracurricular activities provide the same benefits and that poorly structured or weakly supervised activities may not foster positive psychosocial outcomes (Mahoney et al., 2005; Vandell et al., 2022). Future studies should therefore explore how the qualitative features and participation intensity of extracurricular programs interact with school-level learning and instructional conditions.

A further limitation concerns the operationalization of the outcome variable. Although PBABS includes peer intimidation among its components, it also captures broader school-level aggressive and misconduct behaviors such as profanity, vandalism, theft, physical injury caused by students to other students, and intimidation or verbal abuse directed toward teachers or non-teaching staff. Therefore, the findings should be interpreted as reflecting a broader construct of aggression- and misconduct-related school behavior rather than a narrow focus on bullying alone. For this reason, bullying-specific conclusions and implications should be interpreted with caution. Future research should test whether the observed pattern holds when bullying is operationalized using more focused outcome indicators.

Fifth, the main inferential analyses were performed using a complete-case approach based on listwise deletion, thereby reducing the analytic sample relative to the full dataset. Although this method ensured comparability across the correlation, regression, and conditional process analyses, it may have introduced bias if the excluded cases differed systematically from those included in the final sample. In studies with substantial missing data, complete-case analysis can reduce statistical efficiency and yield biased estimates if the missing-data mechanism is not fully random (Enders, 2010; Little and Rubin, 2019). Future research should therefore assess the robustness of the findings using alternative missing-data techniques, such as multiple imputation or full-information maximum likelihood, and include sensitivity analyses across different analytic samples.

Finally, the decision not to use PISA sampling weights in this study limits the extent to which the results can be applied broadly. While unweighted data is appropriate for testing theoretical models and structural relationships, it may not fully account for differences in selection probabilities across groups. As a result, the findings should be seen as reflecting relationships within the sample studied rather than as nationwide estimates. Furthermore, although Country Fixed Effects were explicitly included in the current analyses to account for within-country dependence, the hypothesized model was tested using a single-level conditional process framework rather than a full multilevel modeling (MLM) approach. While the fixed effects approach successfully controls for unobserved country-level heterogeneity, it does not dynamically model the hierarchical structure of schools nested within countries or allow for the estimation of cross-level interactions. Future research could improve the interpretation of cross-national patterns by employing multilevel modeling approaches that better represent nested educational data structures (Raudenbush and Bryk, 2002). Future research aiming for population-level generalizations should also include sampling weights to address this limitation.

## 5 Conclusion

This study provides a contextual contribution to the literature on peer bullying and aggressive behavior by showing that extracurricular activities are not inherently protective. Instead, their connection to bullying-related outcomes seems to work through a moderated mediation model. Specifically, extracurricular activities are indirectly linked to peer bullying and aggressive behaviors through issues that impede the school’s instructional capacity, while the direct link varies with the extent of the obstacles affecting students’ learning. By incorporating issues that limit instructional capacity and factors that hinder students’ learning into a social–ecological moderated mediation model, the study shifts focus from individual behaviors to the institutional conditions that shape students’ daily psychological experiences at school. The findings highlight that bullying should be viewed not just as a behavioral problem but as an institutional and relational issue rooted in the organization of schooling itself. Schools with weak instructional capacity and widespread learning obstacles may struggle to translate extracurricular activities into meaningful psychosocial benefits. On the other hand, when extracurricular activities are part of supportive instructional structures and engaging learning environments, they can help create a school culture that promotes belonging, emotional security, and prosocial norms.

From a broader view, this study emphasizes the importance of moving away from fragmented approaches that treat bullying, extracurricular activities, and instructional quality as separate areas. Instead, it advocates integrated, school-wide strategies that recognize how learning environments, institutional capacity, and student engagement work together to shape peer relationships. By situating extracurricular activities within a broader social–ecological and school psychology framework, the current study contributes to a more detailed, theoretically sound understanding of bullying prevention across various educational settings.

## Data Availability

Publicly available datasets were analyzed in this study. This data can be found here: https://www.oecd.org/en/data/datasets/pisa-2022-database.html.
